# Supplementary material for: Preliminary Study on the Purity Analysis of Primary Certified Gas Mixtures Using Different Spectroscopic Techniques
Source: Sensors (Basel). 2025 Oct 2;25(19):6068. doi: 10.3390/s25196068 (PMC12526893; doi:10.3390/s25196068)
Supplement: Supplementary file 1 [file sensors-25-06068-s001.zip › sensors-3810702-supplementary.pdf]

SUPPLEMENTARY MATERIAL S1

FTIR parameters

- IR source: mid-Infrared (maximum operational range 10-9600 cm^{-1}, used range 400-4000 cm^{-1})
- Multipass gas cell having internal volume 0,2 L and 2 m optical path
- MCT (Mercury-Cadmium-Telluride) detector
- For the analyses described in the paper, a 1 cm^{-1} resolutiom was used, and a spectra averaging time of 224 s (83 scans)

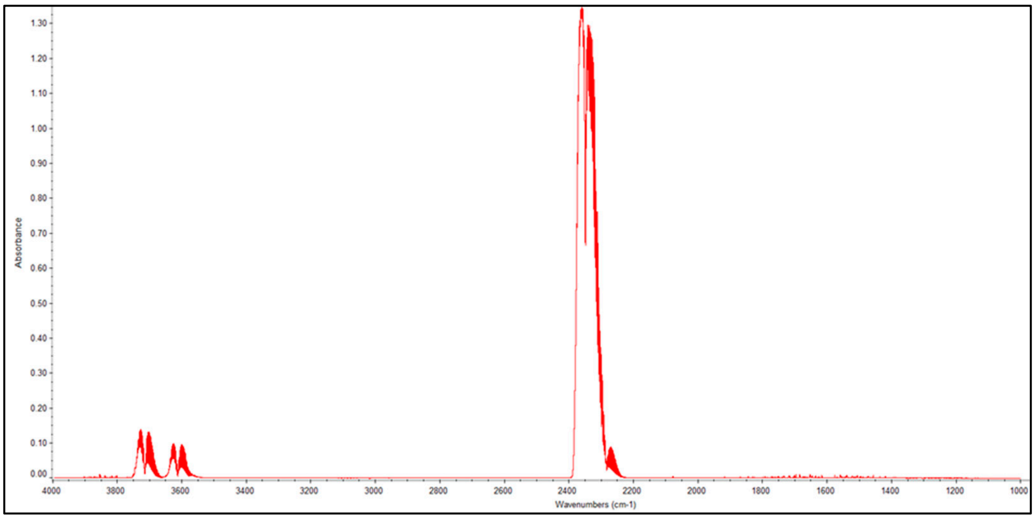

Figure S1: Example of FTIR spectrum of CO_2 in synthetic air, at a nominal amount fraction of 400 μmol·mol^{-1}, obtained with the above mentioned parameters.

NDIR parameters

- NDIR measures individual spectral features, with no interference from other gas species commonly found in ambient air.
- Non-dispersive infrared analyzer exploits a very strong absorption of CO_2 at 4.26 μm and H_2O at 2.59 μm
- Narrow bandwidth optical filters are located in front of the detector, to eliminate all light except the wavelengths in the selected region (Crosson, 2008)

CRDS parameters

- The Picarro analyzer uses wavelength-scanned cavity ringdown spectroscopy technology (Crosson, 2008; Rella et al., 2013; Chen et al., 2013).
- For CO_2 and H_2O: telecom-grade distributed feedback (DFB) lasers.
- One laser measures a single CO_2 spectral feature at a wavelength of 1603 nm while the other measures H_2O (and CH_4) spectral features at 1651 nm.
